# Text message reminders for visit adherence among non-communicable disease patients in Haiti: A pilot study

**DOI:** 10.1371/journal.pgph.0004376

**Published:** 2025-04-17

**Authors:** Calixte Dawson, Sarah M. Morris, Evyrna Toussaint, Darius Léopold Fénelon, Bolanle Banigbe, Gene F. Kwan

**Affiliations:** 1 Zanmi Lasante, Croix-des-Bouquets, Ouest, Haiti; 2 Department of Medicine, Boston University Chobanian & Avedisian School of Medicine, Boston, Massachusetts, United States of America; 3 Boston University School of Public Health, Boston, Massachusetts, United States of America; 4 Section of Cardiovascular Medicine, Boston Medical Center, Boston, Massachusetts, United States of America; 5 Partners In Health, Boston, Massachusetts, United States of America; Tribhuvan University Institute of Medicine, NEPAL

## Abstract

Clinic appointment compliance is a challenge to care continuity for people with chronic non-communicable diseases (NCD) globally. Short-message-service (SMS) text reminders have improved attendance in several settings but have not been tested in Haiti. This pilot study of an SMS reminder strategy to improve clinic attendance for NCD patients in Central Haiti assessed the feasibility and acceptability the messages. We included patients in the NCD clinic – adults with hypertension, type 2 diabetes, and heart failure; and children/young adults with type 1 diabetes (T1D) – at Hôpital Universitaire de Mirebalais. Patients with appointments between December 13-22, 2021, were sent SMS reminders 3 days and 1 day before their visit. Patients completed a survey at their appointment evaluating the usefulness, acceptability, and feasibility of the reminders. We assessed barriers to receiving the SMS and how they influence clinic attendance. Ninety-six patients had a scheduled appointment during the study period. SMS reminders were sent to all patients with recorded phone numbers (91.2%). 72 patients (75%) attended their visit. Half of those who attended their visit received the SMS. Of these patients, 100% liked the reminder, 97.2% wanted to receive future messages, and 22.2% reported they would not have attended their visit without the reminder. Of the 36 patients who did not receive the SMS, 38.9% changed their phone number while 33.3% did not have access to a phone. Barriers to receiving the SMS were inconsistent cellular signal (25%) and lack of access to a phone (22%). Sending SMS reminders was feasible and acceptable to NCD patients. The messages were universally liked by the patients and positively influenced the decision of some to attend their visit. Barriers to uptake include updating phone numbers and access to phones. If implemented at scale, SMS reminders may improve appointment attendance in rural Haiti for those with mobile phones.

## Introduction

Non-communicable diseases (NCDs) including cardiovascular diseases, diabetes, and respiratory illness are responsible for over 70% of deaths worldwide [1]. These conditions are highly prevalent in Haiti and other low-and-middle-income countries (LMICs) [2,3]. The burden of NCDs weighs disproportionately on LMICs, where 80% of NCD deaths occur [4]. Living with an NCD in an LMIC like Haiti increases risk of impoverishment and catastrophic out-of-pocket spending on healthcare [5,6].

Effective management of chronic NCDs requires long term adherence to outpatient clinic visits and medications to prevent complications. Non-adherence to care is a challenge to NCD management globally and has been shown to adversely affect health outcomes [7–9]. Over time non-adherence to appointments, and resultant inconsistent access to medications, contributes to increases in both disease severity and related cost [5,10]. Among patients with heart failure at Hôpital Universitaire de Mirebalais (HUM) in rural Haiti, only 36% returned to the clinic for timely care [11]. Adherence to clinic visits among adults with NCDs at HUM is highly irregular, characterized by missed visits and gaps in care. Only 10% of patients with NCDs meet all studied visit adherence metrics: visit constancy (≥1 visit every 3 months), no gaps in care (> 60 days between visits), ≥1 visit in the last quarter, and ≥6 visits per year [12].

Barriers to appointment adherence occur at the level of the patient, the health system, and the environment. Patient-level barriers include forgetting the appointment, low understanding of chronic NCD care, having a weak personal support system, and work or family obligations that compete for priority [13–15]. Health system barriers include long wait times at the clinic and confusing follow up instructions. Commonly-reported environmental/structural barriers include low availability or reliability of affordable public transportation and having to travel a far distance to the clinic [16].

Many reminder systems have been implemented to address forgetfulness, namely short-message-service (SMS) text reminders [17–25]. A low-cost and easily scalable intervention, SMS reminders have contributed to increased rates of appointment attendance in multiple settings and are found to be acceptable by study participants [22–25]. In LMICs, SMS-based interventions have been shown to improve clinical outcomes like medication adherence and perinatal mortality [18,19]. Other studies have shown that receiving SMS reminders to take medication have helped patients feel that their clinic cares about them, and have led to improved attitudes towards their condition [20,21]. For some people living in rural settings, mobile phones and SMS messages represent the most effective way to connect with the local healthcare system [26].

However, in LMIC settings, there are unique challenges to implementation of mobile health (mHealth) interventions that include frequent changes in phone numbers, volatile telecom sectors, inconsistent access to electricity for device charging, and low digital and health literacy particularly among elderly patients [27,28]. Additionally, the process of adapting intervention material to be culturally and socially congruent to the implementation setting can be lengthy and involves several parties [27].

Despite the increasing utilization of mHealth interventions in LMICs, SMS reminders for appointment attendance have not been adapted for, or implemented in, rural Haiti for NCD care. Due to a paucity of implementation data regarding scaled-up SMS-based interventions in LMICs [29], a feasibility assessment specific to our study setting in Haiti is essential.

In this study, we assess the feasibility and acceptability of a pilot SMS intervention for outpatient clinic appointments for patients living in rural Haiti with an NCD. In this setting, a low-cost intervention able to quickly reach patients who live far from their clinic could be an acceptable and effective way to address the barrier of forgetfulness and improve appointment compliance.

## Methods

We conducted a study of a planned programmatic intervention. This study was conducted in the outpatient NCD clinic at Hôpital-Universitaire de Mirebalais (HUM) in December of 2021. HUM is Haiti’s teaching hospital and serves a catchment area of 1.3 million people in the Central Plateau. The clinic leadership developed and tested the SMS intervention. We then conducted surveys among patient participants, after informed consent, to assess acceptability.

This study included patients from HUM’s outpatient NCD clinic who had a scheduled appointment during the 2-week study period of December 13 – 22, 2021. The clinic serves patients with hypertension, diabetes, heart failure, asthma, and sickle cell disease. All HUM NCD clinic patient with a scheduled appointment during the intervention period and a phone number in the medical record was eligible for the study. The study period was chosen purposively as historically many patients may miss appointments in December prior to the end of the year holidays. We excluded patients without a phone number in the medical record.

The research team developed an SMS message in Haitian Creole to remind the patient of their upcoming appointment. The messages were personalized with the patient’s first and last name to promote engagement [30,31], but did not include further details about the appointment to maintain confidentiality as many patients in Haiti share phones. As many people in Haiti have similar first names, we included the last name to better reach the intended recipient. The message was tested with four NCD patients to ensure comprehension and appropriateness. The SMS reminders were sent at various times between 8:00 AM and 6:00 PM, when a study staff member was available to manually compose and send them. Study staff had a list of study participants and a schedule to track when message should be sent. Messages are free to receive but cost about.0067 USD to send. This cost was paid by the study team.

A sample of the SMS sent to patients: “Good morning [FIRST AND LAST NAME], this is [CLINIC NURSE NAME] at the HUM NCD clinic. You have an appointment on [DAY OF THE WEEK, and DATE]. Contact us at [CLINIC PHONE NUMBER] if you cannot attend or have questions”. The text was sent in Haitian Creole and read: “Bonjou [FIRST AND LAST NAME], se [CLINIC NURSE NAME] nan klinik NCD HUM. Ou genyen randevou [DAY], [DATE]. Kontakte nou nan [CLINIC PHONE NUMBER] si ou pa kapab vini ou byen si ou genyen kesyon’. To increase the likelihood of receipt by the patient, we sent a total of 2 SMS message: one sent 3 days and another 1 day before their scheduled visit. Study participants could reply by text or by phone. Reply text messages are free if they are within the same network, but incur a fee if on a different mobile provider. The texts did not include any content that requested or implied the participants respond to the SMS.

Patients who attended their appointments completed a survey administered orally on the same day as their appointment. The survey was developed to assess the feasibility, acceptability, and usefulness of the SMS reminders. The survey is available in the Supplement (S1 Text). The survey also contained short answer questions wherein patients reported challenges they experienced with receiving text messages as well as described how receiving the SMS contributed to their appointment attendance. Patients who missed their appointments received two phone calls over three days from study staff to confirm receipt of SMS and ascertain reasons for missing the appointment.

Demographic data including biological sex, date of birth, and medical comorbidities were collected from the medical record for all patients with scheduled appointments during the implementation period.

In this pilot study, our primary objective was to assess acceptability to patients, and fidelity/feasibility by the implementers of the SMS intervention. Thus, we assessed selected outcomes related to intervention implementation. We simplified component questions of the Acceptability of Intervention Measure [32]. The acceptability of the intervention was assessed among the patients who reported receiving the message. We asked patients if they liked the reminder and were interested in receiving future reminders. Fidelity was defined as delivery of the intervention as intended [33]. We calculated the proportion of SMS messages sent as planned – three days and one day before the patient’s appointment. Feasibility was defined as the proportion of patients who reported receiving at least one SMS message before their clinic appointment. We did not specifically assess the effectiveness on visit adherence pre- vs. post-intervention in this pilot study. We also estimated the implementation cost to the healthcare system of sending an SMS on local networks as well as the time spent by study staff to compose and send the message. We did not measure client costs to respond to messages or for the cost of transportation or other related expenses to come to the scheduled clinic visit.

Descriptive statistics are reported for demographic characteristics of eligible and enrolled study participants and appointment adherence. We used a Chi square, or Fisher’s exact test where appropriate, to determine whether receiving the SMS reminder was related to phone ownership or cell provider. The same tests were used to determine whether reliance on the SMS reminder for appointment attendance was related to clinic type and age. Short answer responses were grouped into themes and reported with descriptive statistics. Aggregate analysis of all participants was performed, as well as sub-analyses of the T1D and “General NCD” clinic patients. T1D patients arrive on a disease specific day, and compared to the General NCD patients, the T1D clinic has many pediatric patients who often attend appointments with their parents and may be less likely to own their own phone. Due to anticipated differences in phone ownership and patterns of communication with the clinic, the two clinic groups were sub-analyzed separately.

### Ethics

This study was reviewed and approved by the institutional review boards of Zanmi Lasante in Haiti (ZLIRB05212023) and Boston University Medical Campus (H-34326). Written informed consent was obtained from adult participants at the time of the clinic visit, after the SMS messages were sent. Written consent was obtained from the parents of participants under age 18 years.

## Results

### Participant characteristics

**Fig 1:**
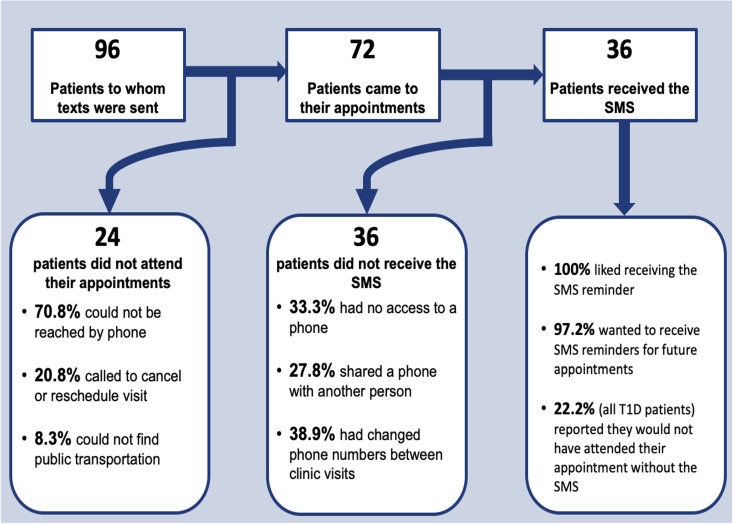
Flowchart describing visit attendance outcomes, descriptive characteristics of patients who did not attend their visit, and an overview of survey results for patients who did.

Ninety-six patients had visits scheduled during the pilot intervention period. Mean age was 48.7 years (standard deviation 20.2) and 63 (65.6%) were female (Table 1). Of all the patients, 36 (37.5%) had hypertension alone, 30 (31.3%) had type 1 diabetes, 6 (6.3%) had type 2 diabetes alone, and 23 (24%) had both hypertension and type 2 diabetes. Seven patients (7.2%) had other diagnoses including asthma, heart failure, stroke, sickle cell disease, and coronary artery disease. Six subjects were under age 18 years – all with type 1 diabetes. At the facility, patients are grouped by similar conditions into a General NCD patient clinic (n=73, 76.0%), and Type 1 diabetes clinic (n=23, 24%) (Fig 1).

**Table 1:** Characteristics of the study sample separated by clinic type: Type 1 Diabetes (T1D) versus other noncommunicable disease (NCD).

	All (n = 96)		General NCD patients (n = 73)		T1D patients (n = 23)	
	Attended visit (n = 72)	Did not attend visit (n = 24)	Attended visit (n = 56)	Did not attend visit (n = 17)	Attended visit (n = 16)	Did not attend visit (n = 7)
Age, mean years (SD)	49.5 (20.8)	46.2 (18.2)	58.2 (14.1)	55.7 (9.0)	18.6 (4.3)	19.2 (2.1)
Patients under 18 years, n (%)	5 (6.9%)	1 (4.2%)	0	0	5 (31.3%)	1 (14.3%)
Female sex, n (%)	42 (58.3%)^†^	21 (87.5%^†^	36 (64.3%)	15 (88.2%)	6 (37.5%)^†^	6 (85.7%)^†^
Chronic disease diagnoses						
Hypertension and Type II Diabetes	19 (26.4%)	4 (16.7%)	19 (33.9%)	4 (23.5%)	0	0
Hypertension alone	26 (36.1%)	10 (41.7%)	26 (46.4%)	10 (58.8%)	0	0
Type II Diabetes alone	4 (5.6%)	2 (8.3%)	4 (7.1%%)	2 (11.8%)	0	0
Type I Diabetes	23 (31.9%)	7 (2.9%)	0	7 (41.2%)	16 (100%)	7 (100%)
Asthma	2 (2.8%)	1 (4.2%)	2 (3.6%)	1 (5.9%)	0	0
Heart Failure	0	1 (4.2%)	0	1 (5.9%)	0	0
Other ^*^	3 (4.2%)	0	3 (5.4%)	0	0	0

*stroke (1), sickle cell (1), and coronary artery disease and anemia (1)

† p < 0.05

There was no significant difference in age between patients who attended and missed their appointments. Female sex was significantly associated with appointment nonadherence for all patients (Chi square p = 0.009) and for T1D patients (Chi square p = 0.033), but not General NCD patients. Among General NCD patients, there was no difference in diagnoses of patients who attended and missed their visits.

### Intervention implementation

#### Fidelity and feasibility.

Of the 96 total patients, 88 (91.7%) had available phone numbers (Table 2). We sent SMS reminders to all the 88 patients with an available phone number 3 days and 1 day prior to their scheduled visit. Of the 72 patients (75% of those scheduled) who came to their visit, 36 (50%) reported receiving the SMS reminder. Thirty-one of 36 patients (86.2%) received the SMS as planned, while four patients (11.1%) reported receiving the message 2 days prior to the visit, and one patient received it the same day as the visit. Twenty-four patients (25.0%) missed their appointment, 17 (17.7% of the total) of whom could not be reached by study staff. Seven patients called beforehand to cancel or reschedule. Two of the patients who canceled their visit cited reasons for cancellation including lack of transportation and concern for personal safety due to ongoing political instability.

**Table 2:** Implementation and appointment attendance outcomes organized by clinic.

	All	General NCD Clinic	T1D Clinic
	(n = 96)	(n = 73)	(n = 23)
**Fidelity**			
Patients who were sent SMS (of all with scheduled appointments)	88/96 (91.7%)	65/73 (89%)	23/23 (100%)
**Appointment attendance**			
Appointments attended (of all scheduled patients)	72/96 (75%)	56/73 (76.7%)	16/23 (69.6%)
**Feasibility**			
Patients reported receiving SMS (of those who attended the appointment)	36/72 (50.0%)	25/56 (44.6%)	11/16 (68.8%)
**Acceptability** survey among the participants who attended appointment and reported receiving SMS			
SMS approval: “Did you like the messages?”	36/36 (100%)	25/25 (100%)	11/11 (100%)
Desire for future SMS reminders: “Would you like to receive SMS reminders for future clinic appointments?”	35/36 (97.2%)	25/25 (100%)	10/11 (90.9%)
Would not have come to appointment without SMS	8/36 (22.2%)	0/25 (0%)	8/11 (72.7%)

#### Appointment attendance.

Sixty of the 72 patients (83.3%) who attended their appointment either owned or had access to someone else’s phone (Table 3). Of the 60 patients who both attended the visit and had access to a phone, 36 (60%) received the SMS. The 12 patients who attended the visit but did not have access to a phone reported not receiving the reminder. Of the patients who had phone access, one-third shared the phone with a spouse, parent, or child. Of the patients who had access to a phone, sharing it with another person was not associated with receiving the SMS reminder (14/21 share vs. 22/39 own, Chi square p = 0.439). Six participants (8%) changed their phone number within the last 3 months, which was not associated with receiving the SMS (2/6 changed number vs. 34/66 not changed number, Fisher’s exact value = 0.674). Digicel was the most popular cell service provider (45%), while 18% used Natcom and 16.7% used both providers. Service provider was not significantly associated receiving the SMS reminder (16/33 Digicel vs. 10/13 Natcom vs 9/12 Both, Chi square p = 0.105). Participants identified challenges to receiving an SMS with short answer responses. Prominent challenges included difficulty charging the phone, low signal, and not having a phone. Among the General NCD patients only, low literacy and smartphone unfamiliarity were reported barriers to receiving the SMS.

**Table 3:** Mobile phone ownership and patient-identified barriers to receiving SMS messages, among patients who attended their clinic visit.

	All (n = 72)	NCD Clinic (n = 56)	T1D Clinic (n = 16)
Do you have phone access?			
Own phone	48 (66.7%)	36 (64.3%)	12 (75%)
Access to someone else’s phone	12 (16.7%)	9 (16.1%)	3 (18.8%)
No phone	12 (16.7%)	11 (19.6%)	1 (6.3%)
Share phone with others			
No	50 (69.4%)	39 (69.4%)	11 (68.8%)
Parent	8 (11.1%)	4 (7.1%)	4 (25%)
Child	9 (12.5%)	9 (16.1%)	0
Husband/Wife	5 (6.9%)	5 (8.9%)	0
Neighbor	0	0	0
Changed phone number in past 3 months	6 (8.3%)	2 (3.6%)	4 (25%)
Participant-reported challenges to receiving SMS reminders			
No challenges	27 (37.2%)	22 (39.3%)	5 (31.3%)
Charge	10 (13.8%)	7 (12.5%)	3 (18.8%)
Signal	15 (20.8%)	8 (14.3%)	7 (46.7%)
No phone	16 (22.2%)	14 (25.0%)	2 (12.5%)
Can’t read messages/operate phone	10 (13.8%)	10 (17.9%)	0

#### Acceptability.

Forty patients reported receiving the SMS; four (4.2%) of which called to cancel their visit beforehand. Of the 36 patients who both received the SMS and came to the visit, 100% reported liking the reminder and 97.2% indicated they would like to receive reminders for future appointments. Short answer descriptions of how the SMS contributed to appointment attendance included motivation to attend their appointment (52.8%), a helpful reminder (44.4%), and indicating the hospital cares about the patient (6.3%). Eight (22%) of the 36 participants who received the SMS reported they would not have attended their appointment without the reminder. All of the participants who reported they would have missed their appointment without SMS reminder were T1D patients.

#### Cost.

Two SMS messages were sent to each patient ahead of their visit. Eight patients had at least two phone numbers in the record and SMS reminders were sent to both. Ninety-five SMS messages were sent on day one and day three, 190 messages in total. SMS cost was between 1-1.50 gourdes (0.0067 USD) per message depending on the length of the patient’s name, paid for by the study team. It took about one minute to send each SMS which amounted to 3 hours and 10 minutes during the 1-week intervention.

## Discussion

We found that our pilot SMS-based intervention to improve adherence to clinic visits was feasible, acceptable, and could influence patient behavior. We sent messages at low cost and many patients received them, despite the barriers identified by the patients. A vast majority of the patients who received messages liked them and wanted future messages. Though this pilot study was not designed to test the effectiveness of SMS messages on visit adherence, nearly one-quarter of patients who received the messages said that they would not have attended their appointment without the reminder. We found that nearly three-fourths of patients in the Type 1 Diabetes clinic reported that they would have missed their appointment without the reminder. Yet, none of the General NCD clinic patients reported that they would have missed their appointment. An SMS-based intervention may be part of a scalable strategy to improve engagement among patients with chronic NCDs in low-income countries among patients with phones who can read the messages. Further study is needed to overcome barriers to receiving messages by patients.

Our study’s findings are largely consistent with those of other SMS interventions promoting care adherence. SMS interventions are often feasible and acceptable. The global availability of mobile phones has increased tremendously in recent years, with 46-62% of people in LMICs estimated to have mobile phone ownership in 2022 [34]. Among our patient population in rural Haiti, about one in six did not have access to a cell phone - even through family, friends, or neighbors. Well-established challenges to SMS based interventions such as problems with signal, smartphone familiarity, and inability to charge devices were also reported by participants in this study [35–37]. The effectiveness of SMS-based interventions is limited for patients who do not have access to mobile phones, are not literate, or live in an area with low service coverage [38]. These factors could have contributed to the gap between patients who were sent the SMS and those who received it. Reliance on a technology-based intervention could potentially worsen existing healthcare access inequities, leaving the poor even further behind [39,40]. For this group of patients who do not own or cannot use mobile phones, a community health worker model could be effective in preserving their connection with the health system.

Participants found the SMS intervention acceptable – there was near unanimous approval of the text message and most wanted to receive future reminders. As done in other studies, we personalized the messages with names of participants and clinic staff, and appointment date to emphasize the personal connection between patient and health system [41,42]. In our study settings, patients must attend clinic visits to obtain their needed medications. Thus, if patients do not come to clinic, they cannot be adherent to their medicines. There is mixed data regarding the impact of personalizing text reminders on healthy behavior, with multiple meta analyses finding it led to no improvement in either adherence to medications or disease control [20,43,44]. However, the short answer responses in our survey revealed that patients felt the SMS messages made them feel that the hospital cared about their attendance. Sending a non-personalized reminder could reduce implementation costs but could result in reduced effectiveness if patients are not reminded of the date of their upcoming appointment.

The strengths of SMS-based interventions are well-established and include low implementation cost and potential for patient-specific individualization. In this study we were limited by our inability to determine the shortcomings of the intervention for patients who missed their appointment with no prior notice and were unable to be reached by study staff afterward. Multiple changes could be introduced to future iterations of this intervention to reduce cost and increase relevance for this study setting including modifications in SMS timing, frequency, content, directionality, and automatization of the messages.

The timing and frequency of texts vary widely among other studies using SMS reminders to improve appointment adherence, from daily texts during the week prior to the appointment to a single reminder 24-hours before the visit [45,56]. Currently, there is no consensus on the optimal timing of SMS frequency, which may depend on the population and intervention [44,46]. In our study, we sent 2 messages to increase the likelihood that the patient would successfully receive the message. We do not know how many patients would have required both messages, or if only one message would have been sufficient. Our study identified the challenge of inconsistent cellular service, and therefore sending more than one SMS reminder that are days apart prior to the appointment may be the most appropriate strategy for this setting.

The SMS reminders in this study were intended to be unidirectional as patients were not prompted to respond to the text reminder with their own message. In contrast, SMS interventions utilizing two-way messaging encourage patients to respond confirming receipt of the text, ask a question, or arrange a phone call with study staff [47]. While some individual studies have found two-way text communication between the patient and clinic to be effective in achieving blood pressure control, meta-analysis of similar studies found one-way messaging to be equally as effective and utilized less resources [44,48]. Though not intended in our study design, multiple patients called the clinic to reschedule their appointment after receiving the SMS reminder. Another two-way intervention could incorporate unstructured supplementary service data technology that is already widely used in LMICs for mobile phone-based financial transactions. These systems can be adapted for digital health interventions to be feasible and acceptable [49]. Augmenting our study intervention to include two-way SMS communication could increase cost but may permit patients to report their own barriers to attending clinic in advance of their visit. If not to improve appointment adherence, this may allow for further understanding of the barriers patients face in attending their visits.

In this study, two SMS messages were manually written and sent by study staff prior to each patient’s visit. While the monetary cost of the intervention was low at around 240 Haitian gourdes (1.5 USD) to send messages to 88 participants, the time spent preparing and sending the SMS reminders amounted to nearly ten percent of a standard work week. If implemented at a larger scale, the time required to carry out the intervention could become impractical. Using an automated program to send the reminders could be effective in reducing costs of full-scale implementation [50,51]. In many cases, automated SMS systems can be integrated into the existing electronic medical record and after the initial expense of installation, there is little cost increase with scale up [24]. As the messages are programmed to be sent automatically, the time and training requirement for clinic staff is considerably less.

Other cost-effective automated systems include interactive voice recordings (IVR), which could benefit a larger population of patients by bypassing the barrier of literacy, encountered in this study [51]. Multiple patients reported receiving the SMS but being unable to read the message or navigate their smartphone to open it. Live phone calls could address this barrier as well and have been shown to be effective in improving appointment attendance, though are not cost effective as clinic staff have to place each call [52]. Both IVR-based interventions and live phone calls may be less convenient than SMS, as calls require the recipient to attend to them immediately and texts can be received and read later. Recipients may miss the reminder entirely if they are unable to pick up the call. Unlike a text message, the call cannot be listened to again unless a voicemail is left. Smartphone apps that have been designed to improve medication adherence and blood pressure control are convenient and effective for patients who regularly use smartphones [53,54]. However, app-based interventions may worsen inequities among populations that have either low smartphone penetration, or low technological literacy.

This pilot study had several limitations. First, the small size and design was helpful for studying implementation challenges, but not effectiveness. The established general NCD and diabetes clinics in which we tested the intervention have small sizes and subgroup analysis and any associations should be considered hypothesis-generating. Second, we did not systematically update phone numbers in the EMR prior to this intervention. Though this limited study feasibility, we learned valuable information about what proportion of patients may have outdated phone numbers. Third, we relied on phone calls to patients who did not come to their appointment to ascertain if they did or did not receive the SMS. However, we did not reach nearly three-fourths of such patients by phone. This is not surprising as they likely have no access to a phone or an outdated phone number. Doing home visits was out of the scope of this study. Fourth, we did not know if patients used standard feature phones or smart phones. Finally, association between phone ownership trends and ability to receive the SMS reminder could become significant in a scaled-up intervention, though not observed in this small pilot study.

Our pilot study of SMS-based intervention to improve adherence to clinic visits showed the system was feasible, could be operated with high fidelity, and was highly acceptable to patients. We uncovered barriers of phone access, unreliable signal, and inability to charge devices. SMS reminders could be an important element of a multicomponent strategy to improve clinic attendance in rural Haiti. An optimized SMS intervention in this setting could include multiple text reminders sent in the days prior to the appointment that contain the date and time of the upcoming visit. Further, an automated system for sending SMS reminders is needed for scalability. An equitable strategy may involve a combination of properly targeted SMS reminders to patients who have access to phones and can understand the messages, with other higher-touch interventions such as phone calls home visits to those without phones to encourage adherence to visits.

## Supporting information

S1 Text Supplementary methods and data tables. (PDF)

S1 Data Data file. (CSV)
