# Supplementary material for: Text message reminders for visit adherence among non-communicable disease patients in Haiti: A pilot study
Source: PLOS Glob Public Health. 2025 Apr 17;5(4):e0004376. doi: 10.1371/journal.pgph.0004376 (PMC12005549; doi:10.1371/journal.pgph.0004376)
Supplement: S1 Text — (PDF) [file pgph.0004376.s001.pdf]

SMS Form

Please complete the survey below.

Thank you!

1.1 Non	________________________________<br>(Name)
1.2 Sèks	◯ male<br>◯ female<br>(Sex)
1.3 Laj	________________________________<br>(Age)
1.4 Eske ou te resevwa mesaj klinik lan te voye pou fè w' sonje randevou w' Jodi a?	◯ Wi (Kontinye nan seksyon 2)<br>◯ Non (Kontinye nan seksyon 3)<br>(Did you receive any SMS reminding you of today's clinic appointment?)

Seksyon 2: Akseptasyon ak Itilite mesaj SMS pou rapèl randevou

Section 2: Acceptability and Usefulness of SMS Reminders

2.1 2.1 Kilè ou te resevwa mesaj lan?	☐ Sa fè 3 jou<br>☐ Sa fè 2 jou<br>☐ Sa fè 1 jou<br>☐ Jodi a<br>(When did you receive the message?)
2.2 Eske ou te renmen mesaj la?	◯ Wi<br>◯ Non<br>(Did you like the messages?)
2.3 Si ou pat resevwa mesaj lan eske ou tap vini nan randevou a Jodi a ?	◯ Wi<br>◯ Non<br>◯ Mwen pa konen<br>(Without the message, would you have come to your appointment today?)
2.4 Nan 1-3 fraz, nan ki fason resevwa mesaj SMS la jwe yon wòl nan vini nan radevou ou a Jodi a?	________________________________<br>(In 1-3 sentences, in what way(s) did receiving the SMS message(s) contribute to your attending clinic today?)
2.5 Eske ou ta renmen resevwa mesaj pou fè 'w sonje randevou klinik ou yo?	◯ Wi<br>◯ Non<br>(Would you like to receive SMS reminders for future clinic appointments?)

Seksyon 3: Enfomasyon sou telefòn selilè ak aseptasyon mesaj SMS pou rapèl randevou

Section 3: Mobile Phone Number Details and Acceptability of SMS Reminders
[Complete this section for all patients]

3.1 Eske ou gen yon telefòn?

◯ Mwen gen telefòn pa m'
◯ Mwen gen aksè ak yon telefòn (fanmi/zanmi)
◯ Non, mwen pa gen telefòn
(Do you have access to a phone?)

3.2 Eske ou pataje telefòn ou ak lòt moun? Si wi, ak ki mounn ou pataje li?

☐ No
☐ Paran
☐ Pitit
☐ Mari/Madanm
☐ Vwazen
(Do you share this phone with other people?)

3.3 Ki sèvis telefòn ou itilize?

◯ Natcom
◯ Digicel
◯ Natcom ak Digicel
◯ Okenn nan yo - Mwen pa gen telefon
◯ Mwen pa konen
(Which cellular service provider do you use?)

3.4 Eske ou chanje nimewo telefòn ou nan 3 mwa ki sot pase yo?

◯ Wi
◯ Non
(Have you changed your phone number in the past 3 months?)

3.5 Nou te voye 2 mesaj ba ou pou fè w' sonje randevou ou pou Jodi a; eske ou ta renmen kontinye resevwa mesaj pou fè w' sonje randevou klinik ou yo?

◯ Wi
◯ Non
(We sent you two reminders about today's visit; Would you like to receive SMS reminders for future clinic appointments?)

3.6 Ki difikilte (1-3) ou genyen pou ou resevwa mesaj sa yo?

____
(What are some (1-3) challenges that you face to receive an SMS reminder?)
